# Collective decision-making in microbes

**DOI:** 10.3389/fmicb.2014.00054

**Published:** 2014-03-03

**Authors:** Adin Ross-Gillespie, Rolf Kümmerli

**Affiliations:** Microbial Evolutionary Ecology, Institute of Plant Biology, University of ZürichZürich, Switzerland

**Keywords:** collective decision-making, microbes, cooperation, coordination, social information, phenotypic plasticity, trade-offs, conflicts

## Abstract

Microbes are intensely social organisms that routinely cooperate and coordinate their activities to express elaborate population level phenotypes. Such coordination requires a process of collective decision-making, in which individuals detect and collate information not only from their physical environment, but also from their social environment, in order to arrive at an appropriately calibrated response. Here, we present a conceptual overview of collective decision-making as it applies to all group-living organisms; we introduce key concepts and principles developed in the context of animal and human group decisions; and we discuss, with appropriate examples, the applicability of each of these concepts in microbial contexts. In particular, we discuss the roles of information pooling, control skew, speed vs. accuracy trade-offs, local feedbacks, quorum thresholds, conflicts of interest, and the reliability of social information. We conclude that collective decision-making in microbes shares many features with collective decision-making in higher taxa, and we call for greater integration between this fledgling field and other allied areas of research, including in the humanities and the physical sciences.

## COOPERATION, COORDINATION, AND SOCIAL INFORMATION

Microbes exhibit remarkably diverse and complex social behaviors. Individuals cooperate to form multicellular structures like biofilms (Nadell et al., 2009) and fruiting bodies (Velicer and Vos, 2009; Strassmann and Queller, 2011), to jointly scavenge resources (Harrison and Buckling, 2009), to attack competitors (Riley and Wertz, 2002; Cordero et al., 2012), to hunt in packs (Berleman and Kirby, 2009), to defend themselves against predators (Jousset, 2012) or harsh environments (Davies, 2003) and to move together across surfaces (Weijer, 2004; Kearns, 2010). From an evolutionary perspective, such behaviors can arise and be maintained only when they provide net direct and/or indirect fitness benefits to individuals (West et al., 2006). It follows then that such activities will only be adaptive in certain environments and in certain social contexts – in particular, when the response is coordinated across individuals. Accordingly, we can identify at the proximate level two key requirements for the evolution of collective behaviors. First, there should be intrinsic phenotypic flexibility in the behaviors of individuals and groups in response to changing social and environmental conditions and, second, there must exist mechanisms to facilitate coordination among members of the collective. Empirical work shows that microbes can indeed flexibly and quickly adjust their phenotypes in response to prevailing environmental and social conditions (Kümmerli et al., 2009b) and most microbial cooperative activities are indeed undertaken in a coordinated manner (Jacob et al., 2004), and only when it is adaptive to do so (Darch et al., 2012; Dumas et al., 2013). Such observations raise important questions: how do simple brainless microbes, living as they do in countless multitudes, achieve such impressive coordination? How do they integrate complex information and reconcile potential conflicts of interest, to arrive at – and then enact – collective “decisions”? Although we have only relatively recently begun to consider these questions in a microbial context, there exists already a sizeable literature in the natural and social sciences on the general topic of collective decision-making in animals and humans. Here, we highlight some general concepts and results from this literature that are relevant to the study of collective decision-making in microbes.

Before we continue, we need to clearly define what is meant by “collective decision-making,” since, despite the large body of literature on this subject, a standard definition is sadly lacking. We define collective decision-making in its broadest sense as the process by which a group of individuals uses social information to arrive at a state of adaptive group-level coordination. By “social information” we mean signals and/or cues generated by other individuals (Maynard Smith and Harper, 2003; Keller and Surette, 2006; Diggle et al., 2007a; Scott-Phillips, 2008), which could be transmitted directly, or indeed indirectly (e.g., through traces left in the environment; i.e., stigmergy). By “group-level coordination” we mean anything other than a random distribution of individuals – or their behaviors – in space or time. It is important to note that group-level coordination could involve either positive or negative correlations across individuals (Giuggioli et al., 2013). Either pattern can potentially provide benefits. Positive correlations of individuals or their activities can lead to greater efficiency and synergy (Sumpter and Pratt, 2009), while negative correlations can minimize mutually harmful or wasteful competition (Giuggioli et al., 2013). Thus, geese flying together in a V-formation (positive correlation in space and time), fireflies spacing themselves evenly in the undergrowth (negative correlation of individuals in space), wolves howling in unison (positive correlation in time) and meerkats taking turns to babysit their group’s pups (negative correlation in time) all represent instances of adaptive coordination, arrived at by some process of collective decision-making.

So, coordination can be adaptive, and collective decision-making uses social information to achieve this coordination. But why use social information to achieve coordinated responses to the environment? After all, patterns of correlation can also arise when individuals respond only to environmental cues only. For example, when phototrophic bacteria sense and swim along light gradients, this can result in striking aggregation patterns at the level for the collective, yet social information may play little or no role in the process (Taylor et al., 1999; Armitage and Hellingwerf, 2003). Yet, there are important advantages to using social information. First, compared to direct first-hand information, social information can potentially be acquired quicker and at lower cost. For instance, individuals in groups that share social information may detect a lurking predator earlier than would solitary individuals (“many-eyes” hypothesis; Lima, 1995). Second, it has long been appreciated that averaging over multiple independent estimates can lead to a more accurate overall estimate (e.g., “wisdom of the crowds”; Galton, 1907). Thus, individuals with access to social information can potentially obtain a more accurate picture of prevailing ecological conditions, and such groups can realize more “accurate” collective decisions – i.e., outcomes that enhance the inclusive fitness benefits to all group members.

## CATEGORIZING COLLECTIVE DECISION-MAKING

**FIGURE 1:**
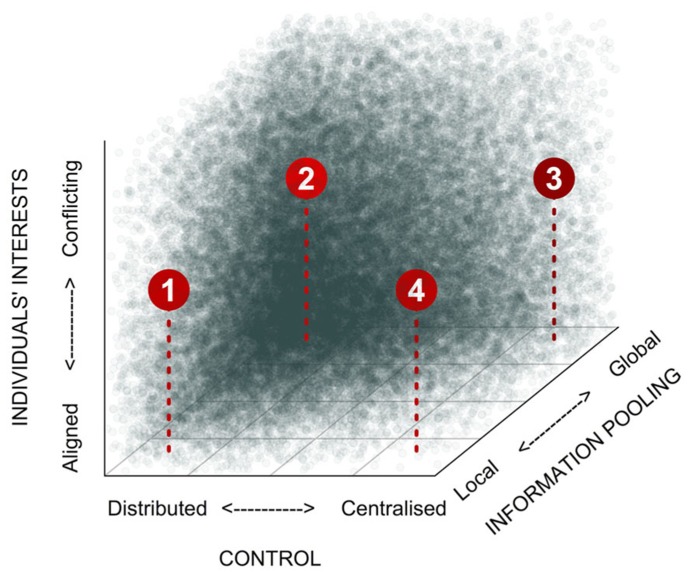
**Categorization of different forms of collective decision-making.** Social settings can differ dramatically with respect to how skewed control is, the scale over which information is pooled, and the potential for conflicts of interests. Shading indicates the expected stability of collective decision-making processes within this parameter space. (1) *Self-organization* (examples: collective movements in bird flocks, fish shoals, ungulate herds, locust swarms; organization and division of labor in social insects or bacterial biofilms); (2) *Democracy* (examples: various human groups, certain primate groups), (3) *Informed autocracy* (examples: hierarchical animal societies, such meerkats or wolves; also certain primate groupings, and human military organizations). (4) “*Blind” autocracy* (few examples from natural systems; unlikely to remain stable over evolutionary time).

Collective decision-making can take many different forms. Building on the concept developed by West and Bergstrom (2011), we propose that three axes delineate the main proximate features of this diversity: (1) the scale over which decision-relevant information is pooled prior to or during decision-making, (2) the degree to which control over decisions is skewed within the group, and (3) the degree to which there are conflicts of interest over the decision outcome (**Figure 1**). The scale over which information is pooled can vary from the global scale, where inputs from all individuals in the group are integrated during decision-making, down to the local scale, where integration of inputs occurs among immediate neighbors only. Control over decisions, meanwhile, can vary from centralized control, where, in the extreme case, a single totalitarian leader determines group behavior, through to completely distributed control (egalitarianism), where each individual has an equal influence on the decision outcome. There is an obvious interaction between the scale over which information is pooled and the degree to which control over decision-making is centralized: highly skewed decision-making is unlikely to be stable unless decision makers have broad access to decision-relevant information (**Figure 1**; West and Bergstrom, 2011). The stability of decision-making processes is further influenced by conflicts of interests among individuals. Along this axis, individuals’ interests can vary from being in complete alignment (e.g., in clonal groups) to being diametrically opposed. In cases where there are conflicts of interest, more egalitarian decision-making and/or broader pooling of information both improve robustness of collective decision-making. In addition, all three variables can typically correlate with group size (King and Cowlishaw, 2007). Global information pooling is more easily achieved in small groups, although sophisticated means of information sharing can make it possible in larger groups too (e.g., humans; McGrath and Hollingshead, 1993). Similarly, logistic constraints can make it difficult to maintain highly skewed control in large groups (Reeve and Emlen, 2000), although multi-tier hierarchies go a long way toward maintaining the skew (Reeve and Keller, 2001; Summers, 2005). Finally, larger groups are also more likely to feature conflicts of interest (e.g., Conradt and Roper, 2007; Brückner, 2010).

Set against this framework, we would predict that decision-making in microbes, which have limited cognitive abilities and usually live in large groups, should be mainly characterized by processes involving local information pooling and distributed control, while the level of conflict of interests would vary from low (e.g., in clonal groups) to high (e.g., in mixed-species communities). In such self-organized decision-making, individuals monitor (chemically) their immediate environment and the activities of their close neighbors only, and respond according to simple innate rules. Taken together, these local interactions can lead to impressive self-organized patterns at the group level. So, what sort of simple innate rules might individuals be following? Couzin et al. (2002) provide an example, based on work by Reynolds (1987). In computer simulations involving groups of continuously moving agents in 3D space, they show that just three rules, applied at the level of individual agents, could lead to the emergence of cohesive, directed patterns of movement at the group level. Specifically, agents monitored three concentric zones centered on their position. If a neighbor entered the outer “zone of attraction,” rule 1 stipulated that they would move toward it. If this neighbor passed a second threshold to enter the “zone of orientation,” under rule 2 they would align to its orientation. Finally, if the neighbor entered the innermost “zone of repulsion,” rule 3 would direct them to move away. Simple sets of rules such as these are thought to underlie the complex collective movement patterns observed in fish shoals (Couzin, 2009), bird flocks (Ballerini et al., 2008), and insects (Buhl et al., 2006). Microbial collective movements too, could be mediated by similar rules, although of course the sensory and cognitive processes would likely be rather different from those of higher metazoans! Shklarsh et al. (2011) used the above approach (Couzin et al., 2002) to model collective swarming motility in a population of bacteria, and found that when individuals adjusted their own motion in response to the movements of nearby neighbors, this robustly improved navigation efficiency in complex environments.

In the following sections, we discuss some general features of collective decision-making processes. In each case, we illustrate concepts using appropriate examples, and we highlight their prospective (or known) relevance in microbial contexts.

## SPEED vs. ACCURACY

One key concept to emerge from the study of human and animal decision-making is that there is an intrinsic trade-off between decision accuracy and decision speed (Schouten and Bekker, 1967; Franks et al., 2003; Chittka et al., 2009). For maximal accuracy, decision makers need reliable information on the likely benefits and costs of all potential alternatives. Obtaining this information can potentially require personal sampling of different options, and/or collating information passed on by fellow group members. Thereafter, some sort of computation must be performed, and finally, the decision must be enacted. All of this takes time, and could involve substantial metabolic investment, risks, opportunity costs, etc. Clearly, in some situations, such as impending danger, it is more important that a decision is made quickly, than that it be the “best” of all possible decisions. This speed-accuracy trade-off applies at all stages and scales of decision-making, including collective decision-making. For example, consider the case of bee or ant colonies choosing among prospective sites for relocation of their communal nest. In cases requiring an urgent decision (e.g., when bad weather is approaching and their current home is compromised), their decision-making process can be fine-tuned to operate faster but less accurately (i.e., the colony may fail to identify the best option and instead may settle for a sub-optimal site). In contrast, when the need to relocate is less pressing, the decision-making process is slower but more accurate (Franks et al., 2003; Pratt and Sumpter, 2006; Visscher, 2007). Note that to achieve maximal accuracy, decision-making mechanisms also need to be flexible. In very changeable environments, what was at first a great option may quickly become a bad option, even while the decision-making is still underway. The best-adapted decision-making processes should therefore allow for some degree of bet-hedging, and also be updatable and reversible (Seger and Brockmann, 1987). Only in this way can groups identify and settle on the current optimal, most accurate, decision. Usually, however, such flexibility features involve additional costs in terms of reduced speed or efficiency. For example, ant colonies face collective decisions about how to deploy their workforce so as to exploit known food sources as quickly and efficiently as possible; yet, at the same time, they must send out scouts to locate new food sources (Latty and Beekman, 2013).

**FIGURE 2:**
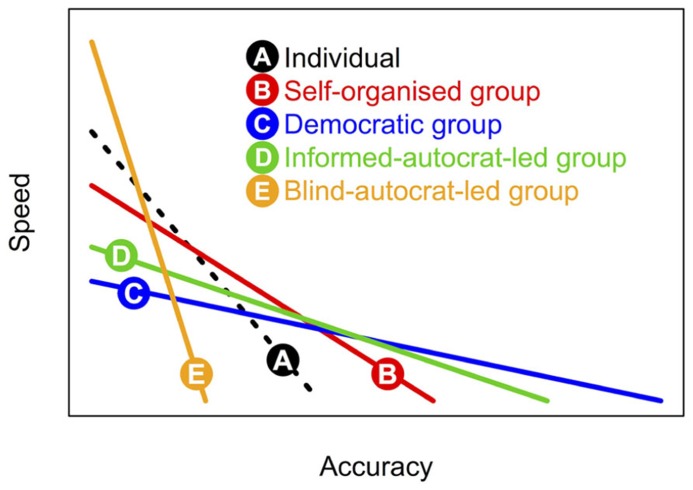
**Speed-accuracy trade-offs under different forms of decision-making.** Different forms of decision-making (1–5) are inherently variable in terms of accuracy (or fairness). For the above illustration we assume the pattern C > D > B > A > E with regard to accuracy. Moreover, the different forms require relatively more or less time for (i) investigation of available options; (ii) assimilation of social information, (iii) deliberation/computation, and finally, (iv) implementation. Here, we assumed the following time costs across decision-making forms A–E: for step (i): 3,2,1,1,1; (ii): 0,1,3,3,0, (iii): 0, 3,1,1,0, and (iv): 1,1,2,2,2.

Since different forms of collective decision-making (see **Figure 1**) feature differing patterns of investment in information gathering and integration, the resulting shape of this speed-accuracy trade-off varies too across categories (**Figure 2**). Comparison of these different trade-off functions indicates that although democratic decision-making may lead to the fairest and most accurate outcomes, more autocratic decision-making is an intrinsically faster process, and so may be adaptive when quick decisions are required.

Where extrinsic stresses are highly variable over time and space, individuals and groups will also be under selection to be flexible in their collective decision-making, having the ability to transition quickly between decision modes favoring high accuracy and those favoring high speed. For example, under acute stress, individuals could benefit by temporarily downscaling their investment in the acquisition and transmission of new social information, and rather just copy or conform to the decisions of better informed “leader” individuals (Couzin et al., 2005; Dyer et al., 2009).

To our knowledge, the speed-accuracy trade-off in decision-making has not received much attention in microbes, although we would expect that it should still apply. Certainly, the collective decisions achieved by clonal microbe groups do appear to be impressively flexible in response to environmental changes, and this flexibility has been interpreted as a form of bet-hedging (Beaumont et al., 2009; Ratcliff and Denison, 2010; de Jong et al., 2011; Levy et al., 2012). It is thought that such flexibility arises because individuals stochastically switch between alternating distinct responses (Kussell and Leibler, 2005). Social information may indeed play a role here, but so far this role has not been explicitly considered.

## POSITIVE AND NEGATIVE FEEDBACKS

**FIGURE 3:**
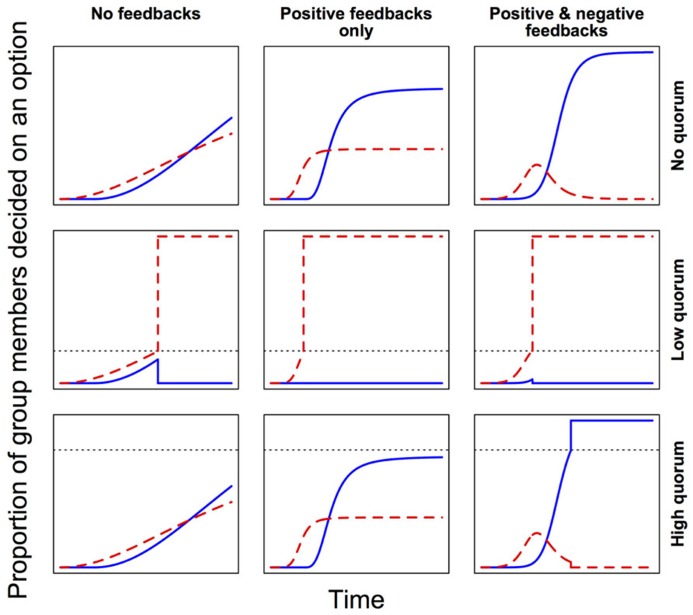
**Feedbacks and quorum thresholds modulate the speed and accuracy of collective decision-making.** Here, groups are deciding between two options. Red (dashed line) is the first option to attract support, but blue (solid line) is the more accurate choice (i.e., leads to higher average fitness). Lower quorum thresholds (dotted lines) make decision-making faster, but increase the risk of suboptimal outcomes. Positive feedbacks reinforce preferences and so accelerate decision-making, but negative feedbacks provide flexibility by avoiding deadlocks and allowing suboptimal choices to gradually lose their appeal. Note that here “quorum” is defined as some critical threshold proportion of decided individuals, above which a consensus decision is immediately effected.

In self-organized decision-making systems, local feedbacks are very important (Sumpter, 2006). During the exchange of social information among neighbors, positive feedbacks lead to amplification of transmitted signals, promoting their accumulation and spread, while negative feedbacks dampen this accumulation and spread of social information. In general, positive feedbacks speed up decision-making, while the addition of negative feedbacks confers flexibility and hence allows for a more accurate response (**Figure 3**). For instance, where groups must decide between discrete alternatives, strong positive feedbacks could quickly drive up support for the first detected (but not necessarily the best) option, while potentially better alternatives remain still undiscovered (Grüter et al., 2012). By slowing the process, negative feedbacks can help to avoid this suboptimal outcome. Also, where two or more alternative options are attracting similar levels of support within a group, negative feedbacks can facilitate the relatively steeper decay of support for less preferred options, and thus avoid deadlocks. Finally, when deciding on some coordinated response from a continuous range of possible alternatives (e.g., when to leave; how much of a substance to produce), negative feedbacks counterbalance positive feedbacks and so allow fine-tuning of the response. In each case then, positive and negative feedbacks work together to modulate the trade-off between the speed and accuracy of the decision-making process. Tweaking the relative strengths of these different feedbacks can thus shift a system toward prioritizing either accuracy or speed (Franks et al., 2003; Pratt and Sumpter, 2006).

Feedbacks are well-established features of decision-making in social insects and other arthropods (Hölldobler and Wilson, 1990). For instance, foraging Pharaoh ants (*Monomorium pharaonis*) lay pheromone trails when returning to their nest from remote food sources (Beekman et al., 2001), and these trails help to direct additional foragers to the food in a positive feedback loop. Foragers adjust their pheromone deposition (and thus feedback intensity) in proportion to the quality of the food source (Jackson and Châline, 2007), allowing the self-organized ants to collectively arrive at a efficient pattern of resource exploitation. Similar positive feedbacks, involving silk draglines, facilitate group aggregation in young spiders (Jeanson et al., 2004). To illustrate the interactions between positive and negative feedbacks in collective decision-making, let’s revisit the example of house-hunting bees mentioned earlier. When colonies outgrow their nests, scouts set off to explore new potential nest sites, and on their return, they communicate their “observations” to other colony members via a special “waggle dance,” in which the duration of the dance correlates with the quality of the visited site. Other bees that perceive this information are recruited to the same site, and in this way, a suitably calibrated positive feedback loop is established (Seeley and Visscher, 2004). However, when multiple similarly suitable sites are available, there is a danger that positive feedbacks alone could lead to a deadlock, such that none of the candidate sites emerges as a clear winner. To avoid this, the decision-making process also includes a negative feedback loop, whereby, in addition to recruiting for the nest site they visited, scouts will also actively disturb the waggle dances of other scouts returning from competing nest sites (Seeley et al., 2011). The combined effect of positive and negative feedbacks in this system means that scouts that visited the most attractive site will recruit the most followers, and most strongly inhibit the dancing of competing scouts, thereby directing consensus decision-making toward choosing the best available nest.

Do such feedbacks also operate in bacteria? Certainly, many bacterial traits, including collective traits, are subject to positive feedbacks (Guespin-Michel and Kaufman, 2001; Smits et al., 2006; Fujimoto and Sawai, 2013). A recent example comes from *Pseudomonas* *aeruginosa* bacteria during the early stages of biofilm formation (Zhao et al., 2013). When bacteria first touch down on a surface, they move about by secreting a film of polysaccharide (Psl) and then pulling themselves over this film using type IV pili. In the process, they leave a trail of Psl behind them, over which subsequent cells can move more rapidly and more easily. This creates a positive feedback effect and leads to local aggregations of cells in much the same way as the pheromone trails and silk draglines described in the arthropod examples above. Similar positive feedbacks operate during the surface expansion of biofilms, where cells closest to the edge leave eDNA-lined furrows as they push outward, which trailing cells then preferentially follow (Gloag et al., 2013).

Negative feedbacks also feature in microbial collective activities. Dumas et al. (2013) studied *Pseudomonas aeruginosa*’s decision to invest in iron-scavenging siderophores as a function of environmental iron availability. Siderophores are shared traits (i.e., public goods) and as such represent a collective action that is most profitable for producers when there is coordination at the group level (Griffin et al., 2004). *Pseudomonas aeruginosa* can produce a potent but expensive siderophore (pyoverdine) that is most beneficial in strictly iron-limited conditions, and/or a weaker but cheaper siderophore (pyochelin) that is the more efficient of the two under less stringently iron-limited conditions. In either case, production is subject to positive and negative local feedbacks (Visca et al., 2007; Youard et al., 2011). Incoming iron-loaded siderophores trigger production of more siderophores (Lamont et al., 2002; Michel et al., 2007), and the strength of this positive feedback correlates with the overall siderophore investment of the local population. At the same time, as more and more iron is taken up, negative feedbacks act to constrain siderophore production (Leoni et al., 2000; Youard et al., 2011). The combination of social information pooling, positive and negative feedbacks, and – in this case – interactions between the two different sets of feedbacks regulating the two siderophores, allows bacteria to individually adjust their overall siderophore “investment portfolio” to match prevailing conditions. Consequently, at the group level, a coordinated and adaptive siderophore investment strategy can be realized. Feedback loops can also facilitate decisions that lead to divergent patterns at the population level, and mediate division of labor. For instance, the aggregation of starved *Dictyostelium discoideum *amoebae is facilitated by a positive feedback loop operating on cAMP secretion (Li and Purugganan, 2011), which establishes a chemical gradient that individual amoebae follow, until a dense aggregation is formed. Later, when the multicellular aggregate commences fruiting body formation, the amoebae must collectively decide how to divide themselves between the supporting stalk and the spore it will bear. Prespore cells toward the front of the aggregate produce a differentiation-inducing factor (DIF-1), which triggers, on the local scale, differentiation of naïve cells into prestalk cells. These prestalk cells, in turn, start to produce an enzyme that cleaves DIF-1, thereby exerting a negative feedback on the differentiation process. Collectively, these feedbacks ensure that just the right number of cells is directed toward the stalk fate.

## THRESHOLDS, QUORUMS AND QUORUM SENSING

Quorums feature in many collective decision-making processes, yet, it is important to note that the term “quorum” has come to mean quite different things across different taxa and in different decision contexts. In human groups, where the term was first used, a quorum is the minimum number of participants required at a meeting before any officially binding collective decisions can be made (Sinclair, 1988). However, when groups of animals make consensus decisions between discrete options, such as shelters, the term “quorum” is used not for the minimum number of participants, but rather the minimum number of committed individuals (i.e., “votes”) for a given option that will swiftly trigger concordant behavior in the rest of the group (i.e., a “quorum response”; Seeley and Visscher, 2004; Ward et al., 2008, 2012; Sempo et al., 2009; Robinson et al., 2011). Consider, as an example, the house-hunting bees example introduced earlier. If, upon arriving at a prospective nest, scout bees detect that around 10–15 of their fellow scouts are simultaneously checking out the same site, then on their return to the swarm, they will produce a specific piping signal that prompts the swarm to prepare for take-off (Seeley and Visscher, 2004). In models describing the collective movement of self-propelling particles (e.g., flocks of birds), meanwhile, we encounter yet another type of threshold: the minimum number of closest neighbors a focal individual must monitor in order to be able to satisfactorily match its own movements to that of its group (Ballerini et al., 2008). Although this particular threshold is not termed a “quorum” (it is “topological distance”), it is certainly a related concept. In microbes, the “quorum” in “quorum sensing” phenomena translates neither to the number of participants in a decision-making process, nor the number of individuals in favor of a particular decision, but rather to a threshold level of stimulus beyond which a standard response is effected. The signals in microbial quorum sensing systems are small molecules, secreted by cells in response to their local conditions, that diffuse freely among cells and bind to intracellular receptors (Waters and Bassler, 2005; Williams et al., 2007; Schuster et al., 2013). This induces the production of additional signal molecules (positive feedback), and, once the signal receptors are sufficiently stimulated, a group-coordinated shift in gene-expression is induced (Schuster et al., 2003). In general, the concentration of signal provides an index of population density, but since cells can vary in the level of signal they produce, this relationship will likely be non-linear in heterogeneous environments or where cells differ in their response to their environment and/or to the social information they receive (i.e., non-clonal populations). Despite this important difference, the resulting outcomes of microbial quorum sensing processes are still comparable, in a qualitative sense, to the “quorum responses” observed in animal groups, in that in both cases they allow behavior to be coordinated at the group level. Accordingly, quorum sensing regulates many cooperative traits that are only adaptive when expressed in a coordinated manner and when the population is at high density, including swarming, exoproduct secretion, and biofilm formation (Nadal Jimenez et al., 2012). One of the classic examples of quorum sensing involves the marine bacterium *Vibrio fischeri* which symbiotically colonizes a specialized light organ of the bobtail squid *Euprymna scolopes* (Ruby, 1996). As it grows to high density in the shelter of the light organ, *V. fischeri* synthesizes (using its LuxI protein) and secretes signal molecules [acyl homoserine lactones (AHLs)], which diffuse among cells and eventually bind to their cognate receptor (LuxR). LuxR ligation stimulates further AHL production (positive feedback) but if no AHL binding takes place, the LuxR protein quickly degrades (negative feedback). When a threshold concentration of LuxR–AHL complex is reached – which, because of the diffusion of AHLs, occurs at roughly the same time in all cells – the luciferase operon is activated and light is produced, in a synchronized manner, by the entire bacterial collective. The light compensates for the squid’s shadow, camouflaging it against the moonlit sky and hiding it – and its bacterial symbionts – from predators in the depths below.

Thresholds can of course also be important in systems that do not employ “recognized” quorum sensing signals like AHLs. For instance, consider the bacterium *Paenibacillus dendritiformis*, which when growing on agar surfaces swarms out to form elaborate fringing colonies that end abruptly at the frontier with another colony, maintaining a well-defined no-man’s land separating the colonies. Be’er et al. (2009, 2010), seeking to explain this group-level coordination, uncovered a relatively simple mechanism operating at a local scale. Metabolically active cells along the colony’s expanding front secrete a protease (subtilisin), which accumulates locally. So long as the colony is expanding outward into virgin territory, subtilisin remains diluted across space. However, upon encountering another conspecific “front,” subtilisin concentrations in the contact zone can build up until a critical level is exceeded, which triggers the localized production and secretion of a bacteriocin (sibling lethal factor (Slf)], that kills cells on both fronts. Consequently, an open zone of inhibition is maintained between the colony fronts. This mechanism is thought to mediate competition over space and resources in a way that is beneficial at the level of the collective.

From the above examples, it becomes clear that microbial quorum sensing systems involve far more than just a quorum. In fact, they involve multiple features of collective decision-making that we have highlighted in this review. First, the diffusion of signals facilitates broad pooling of information. In effect, this moves us along the axis from local to more global information pooling (**Figure 1**). By averaging across multiple potentially noisy informational inputs, decision makers (in this case, individual cells) can build up a richer and more accurate impression of their surroundings and their neighbors (Simons, 2004), and thus arrive at more accurate, more egalitarian, outcomes. Quorum sensing systems also involve an intrinsic response threshold, and – typically – some local feedbacks. Both the response threshold and the feedbacks can be altered, over the short term via phenotypic plasticity, or over the long term via selection, to shift decision-making toward greater speed or greater accuracy (**Figure 3**), to maximize adaptiveness under prevailing conditions (Pratt and Sumpter, 2006). Indeed, when the quorum sensing signal threshold was experimentally lowered in a recent study, and a premature collective response was induced, a fitness drop was observed at the level of the population (Darch et al., 2012).

Although it is now well established that quorum sensing systems provide important benefits through facilitation of cooperative behaviors (West et al., 2012), it is also important to note that they may also provide benefits in non-social settings. For instance, by secreting and monitoring their own signal molecules, individual cells could potentially acquire information about the diffusive properties of their local environment and adjust their own activities accordingly (Redfield, 2002; Hense et al., 2007). Also, some cell products under quorum sensing regulatory control provide little if any benefit to cells other than the producer (Dandekar et al., 2012). More generally then, we may expect to see that mechanisms facilitating collective decision-making could play other roles too.

## CONFLICTS OF INTEREST

Conflicts of interest among individuals will generally destabilize cooperative activities and the collective decision-making processes that facilitate them (as illustrated by the vertical axis in **Figure 1**), but this destabilization depends on the magnitude of the conflict, i.e., the potential fitness advantage an individual could obtain by acting independently (selfishly) vs. coordinating its activity with others (cooperatively). For instance, imagine there is some theoretical level of exoenzyme production that would maximize the final yield of a population of bacteria collectively exploiting a substrate. In principle, bacterial cells could monitor the output of their neighbors, and each could adjust its individual contribution so that the total amount of exoenzyme produced is close to the theoretical optimum, and the costs of producing this required amount are more or less evenly borne by all constituent cells in the population. However, now imagine that within the population, mutants arise that do not coordinate production with others, but rather down-regulate their own contribution to the exoenzyme pool. This lineage would bear a lower cost, and hence should gradually come to occupy a larger proportion of the population. Under this scenario, there could be substantial differences in fitness between individuals contributing their fair share vs. those contributing less than a fair share, so the pattern of coordinated behavior and the decision-making process by which it is achieved would both be quickly undermined by the emergence of free-loading cheats (Giraldeau and Caraco, 2000; Ghoul et al., 2013). Indeed, cheats are frequently observed in numerous microbial cooperative traits, including the shared production of food enzymes (Greig and Travisano, 2004), siderophores (Griffin et al., 2004; Dumas and Kümmerli, 2012), and toxins (Raymond et al., 2012), and also during the formation of multicellular structures like biofilms (Rainey and Rainey, 2003; Popat et al., 2012), and fruiting bodies (Strassmann et al., 2000; Velicer et al., 2000).

To avoid conflicts and maintain collective actions, the interests of group members must be brought into alignment. In general terms, this can be achieved by (a) increasing relatedness, (b) disincentivizing cheating by introducing costs for potential dissenters, or (c) incentivizing cooperation by increasing the benefits associated with cooperation (Hamilton, 1964; Frank, 2003).

(a) Clonal groups have wholly overlapping evolutionary interests, whereas individuals of different strains or species that do not share the same genotype frequently have diverging interests (Foster and Bell, 2012; Rendueles and Ghigo, 2012). Accordingly, experimental work confirms that relatedness is a key promoter of microbial collective actions (food enzymes: MacLean and Brandon, 2008; siderophores: Kümmerli et al., 2009a; Ross-Gillespie et al., 2009; toxins: Inglis et al., 2009; quorum sensing controlled traits: Diggle et al., 2007b; swarming motility: de Vargas Roditi et al., 2013; biofilm formation: Nadell and Bassler, 2011; and fruiting body formation: Gilbert et al., 2007). Nonetheless, natural microbial communities typically involve interactions among individuals that vary widely in their relatedness to one another (e.g., biofilms; Elias and Banin, 2012; Rendueles and Ghigo, 2012) so conflicts of interests are expected to be commonplace in such communities (Xavier and Foster, 2007).
(b) Mechanisms to disincentivize dissention (i.e., repress competition) can take different forms. For essential traits, negative-frequency-dependent selection intrinsically limits the spread of cheats, because at high cheat loads collective actions can collapse – to the detriment of all. Examples here include the collective production of siderophores (Ross-Gillespie et al., 2007), toxins (Raymond et al., 2012), and food enzymes (Gore et al., 2009), as well as fruiting body formation in bacteria (Velicer et al., 2000) and amoeba (Gilbert et al., 2007). In other cases, specific features of the genetic architecture prevent dissention. For example, genes encoding cooperative traits could also pleiotropically influence important personal traits, such that mutants for these genes would not have a net advantage (Foster et al., 2004; Dandekar et al., 2012). Sophisticated regulatory circuits, meanwhile, can ensure that cooperative traits are preferentially expressed when cheating opportunity is low (Kümmerli and Brown, 2010; Xavier et al., 2011). Finally, alignment of interests can also be enforced through mechanisms that sanction or punish cheating individuals (Kiers et al., 2003; Manhes and Velicer, 2011), reward faithful partners (Kiers et al., 2011), or randomize the reproductive success across group members (Kümmerli et al., 2010; Strassmann and Queller, 2011).
(c) Another way in which interests of individuals become aligned is where group coordination leads to massive benefits for everyone. For example, consider a flock of birds migrating from A to B. Although individual birds may have different preferences regarding the timing of the migration or which specific route to take (e.g., depending on their specific size or age, fat reserves, experience, etc.) it is nonetheless important that the birds stay together during their migration so as to benefit from the gains in energy efficiency that can come from slip-streaming behind one another during formation flight. Here, it doesn’t pay for individuals to obdurately pursue their individual optima, so the decision-making process should be more robust. Note, in this example – birds choosing a migration route – it also doesn’t pay for each individual bird to exhaustively assess all its alternative migration options. Considering the fitness payouts at stake here, compromising and simply following others could represent the most rewarding strategy (e.g., Biro et al., 2006; Nagy et al., 2010). Similarly, when individual *D. discoideum* amoebae aggregate in response to starvation, their resultant group-coordinated motility allows everyone to escape hostile conditions (Li and Purugganan, 2011), so there is little evolutionary incentive not to participate, at least in this part of the process.

Besides destabilizing cooperative actions, and the collective decision-making processes that underlie them, conflicts of interest can also influence selection for different forms of decision-making. When conflicts are relatively minor, and where information can be pooled fairly broadly (e.g., small groups) it is easy to see how leaders could emerge – typically from among those individuals for whom different decision outcomes would have strongest effects on fitness – and how a initially democratic decision process could transition into a more autocratic one (**Figure 1**). Where more substantial conflicts of interest exist, meanwhile, democratic systems of collective decision-making should be the most robust, since these give rise to the most accurate decisions (i.e., best-fit compromise across all individuals; **Figure 2**). Conradt and Roper (2010) emphasize that it can be more difficult for groups to reach consensus when deciding between discrete options (since some individuals must wholly abandon their preferred choice, while others will have exactly their preferred choice) than it is for decisions among continuously distributed options (where the consensus can be achieved by simply averaging across preferences) since the latter will generally produce a lower variance between preferred options and final consensus choice. Consequently, conflicts of interest may remain inherently higher in the first case (making democratic decision-making preferable here), than in the second case (where autocratic decisions could more easily evolve). Thus, conflicts of interest can affect decision-making not only in quantitative ways, by making it less robust, but also in qualitative ways too, by selecting for different forms.

## RELIABILITY OF SOCIAL INFORMATION

Incorporating social information into decision-making processes is only adaptive when the incoming information is reliable (Condorcet, 1785; List, 2004). However, individuals will inevitably differ one from another in their detection and responsiveness to such stimuli. Consequently, the amount and reliability of social information will vary across a group. Where some individuals are better informed than others, or are known to transfer this information with greater fidelity, it could be better for all that the decision be made on the basis of their information only, and that the signals and cues from naïve or unreliable individuals be ignored (Reebs, 2000; King and Cowlishaw, 2007; Katsikopoulos and King, 2010). In effect, segregation into informed “leaders” and uninformed “followers” could facilitate a division of labor, with potential gains in efficiency at the group level. Accordingly, when bees are deciding on and relocating to a new nest site, only information from informed scouts who have first hand experience of prospective sites is considered (Britton et al., 2002; Beekman et al., 2006).

In cases with conflict of interest, there is an evolutionary incentive for individuals to distort collective decision-making toward more personally favorable outcomes, and one way to achieve this could be by supplying false or inaccurate information. The evolutionary dynamics of such “dishonest signaling” have been extensively investigated by theoreticians (e.g., Maynard Smith and Harper, 2003; Scott-Phillips, 2008) and could, it is thought, be relevant to microbes. For instance, although inter-individual heterogeneity in producing or responding to quorum sensing signal is typically interpreted as a sort of division of labor that confers group level benefits (e.g., Anetzberger et al., 2009, 2012), it may be that in some cases what we are observing is in fact cheating or coercion, whereby individuals chemically manipulate quorum sensing-based collective decisions to their own advantage, and benefit at the expense of the group (Keller and Surette, 2006; Diggle et al., 2007a; Stacy et al., 2012). Both scenarios are plausible, and to disentangle them we should study fitness consequences at the individual and group level. Certainly, signal-blind quorum sensing mutants are known to arise in natural settings (e.g., the cystic fibrosis lung; Köhler et al., 2009), and *in vitro *experiments suggest that these mutants may be acting as cheats (Diggle et al., 2007b). As another example, during the formation of *D. discoideum *fruiting bodies mentioned earlier, aggregates of amoebae differentiate into prestalk and prespore subpopulations, but some mutants are able to skew the decision-making process so that they end up over-represented in the prespore region of the aggregate, thereby displacing wildtype amoebae to the prestalk region instead (Santorelli et al., 2008).

## OUTLOOK

In this article, we have sought to highlight how some general concepts emerging from the study of collective decision-making in animals and humans can aid in understanding how microbes use social information to coordinate their collective behaviors. We show that microbial collective decision-making shares much in common with collective decision-making in higher taxa, but of course there are some obvious differences too. For example, the typically very large population sizes in microbes could favor mostly distributed decision-making processes as opposed to centralized control. Also, information exchange in microbes is overwhelmingly chemical in nature, whereas in more complex metazoans it may be audial, visual or tactile, etc.

Currently, there is little sign of “cross-talk” about collective decision-making at the microscopic vs. macroscopic scales (although, of course, there are some exceptions, e.g., Weitz et al., 2008; Zeng et al., 2010; Fujimoto and Sawai, 2013; Norman et al., 2013), and it is our hope that this will soon change. In fact, collective decision-making is a phenomenon of even broader relevance. Indeed, collective decision-making mechanisms – featuring local-scale signaling, feedbacks and thresholds – underlie group level organization and coordination not only in humans, animals, and microbial populations (Sumpter, 2006; Conradt and List, 2009; this review), but in many other contexts too. For instance, within the developing tissues of multicellular eukaryotes, a “community effect” facilitates en-masse differentiation of cells upon attainment of some threshold density (Gurdon et al., 1993; Balázsi et al., 2011; Saka et al., 2011). Collective decision-making mechanisms are relevant also in robotics (Garnier, 2011), in cognitive neuroscience (Couzin, 2009; Marshall et al., 2009; Seeley et al., 2011), and in many other contexts, attracting attention from political scientists, economists, ethologists, physicists, mathematicians, computer scientists, neurologists, and urban planners. Clearly, there is scope for much broader dialog on this topic.

In a microbial context, there is still much to be discovered about the mechanisms underlying collective decision-making. While it is clear that microbes share social information and use feedbacks and quorum thresholds to optimize and coordinate social behaviors, other aspects of their collective decision-making are less well explored. For instance, to our knowledge, the speed vs. accuracy tradeoff in collective decision-making has not been explicitly investigated in microbes, although a recent study by Darch et al. (2012) could potentially be interpreted along these lines. Furthermore, while empirical work on human and animal decision-making typically involves monitoring individuals’ behaviors through space and time, microbial experiments typically focus only on behavioral shifts at the population level (although see Gloag et al., 2013; Zhao et al., 2013). For a more complete understanding, microbiologists will need to fully embrace inter-individual variability within these populations – and ideally over short time scales. Thankfully, however, modern technological advances in microfluidics and time-lapse microscopy are making this more feasible (Wessel et al., 2013). In other respects, microbes already offer advantages that animals and humans do not. In particular, we can strictly control the conditions to which microbes are exposed, and we can decode in great detail – and manipulate – the molecular pathways involved in decision-making.

So, what sort of experiments do we think would be most helpful in advancing our understanding of microbial collective decision-making? Let’s consider a hypothetical example. As discussed earlier, it is expected that decision accuracy should improve when information can be pooled across multiple inputs (Sumpter et al., 2008; Ward et al., 2011). One way to test this could be with choice experiments, where, in the simplest scenario, individuals or groups of bacteria are placed in an agar-lined Y-maze and must decide between two nearby alternatives: food or no food. Would larger bacterial aggregates be more likely to swarm in the direction of food sources than smaller groups? Would they “decide” earlier? Would the level of starvation (i.e., the urgency for a collective action) affect the accuracy vs. speed of the decision?

While we do certainly advocate a more interdisciplinary and integrative approach to the study of collective decision-making, at the same time, we urge caution. Social evolution theory has been famously dogged by semantic confusion and controversies (Lehmann and Keller, 2006; West et al., 2007; Nowak et al., 2010; Abbot et al., 2011), and it would be most unfortunate if the same difficulties recurred in this emerging field. The use of clearly defined terminology, and a willingness to engage meaningfully with the work – past and present – from other allied disciplines will be key to the development of general theory for collective decision-making in all its forms.

## Conflict of Interest Statement

The authors declare that the research was conducted in the absence of any commercial or financial relationships that could be construed as a potential conflict of interest.
